# A GdW_10_@PDA-CAT Sensitizer with High-Z Effect and Self-Supplied Oxygen for Hypoxic-Tumor Radiotherapy

**DOI:** 10.3390/molecules27010128

**Published:** 2021-12-26

**Authors:** Lixia Chen, Yang Zhang, Xinming Zhang, Ruijuan Lv, Rongtian Sheng, Ruimeng Sun, Ting Du, Yuhan Li, Yanfei Qi

**Affiliations:** School of Public Health, Jilin University, Changchun 130021, China; chenlx20@mails.jlu.edu.cn (L.C.); yangzhang19@mails.jlu.edu.cn (Y.Z.); xmzhang19@mails.jlu.edu.cn (X.Z.); Lvrj20@mails.jlu.edu.cn (R.L.); shengrt19@mails.jlu.edu.cn (R.S.); Sunrm20@mails.jlu.edu.cn (R.S.); duting21@mails.jlu.edu.cn (T.D.); yhl21@mails.jlu.edu.cn (Y.L.)

**Keywords:** radiosensitizer, polyoxometalates, catalase, O_2_ self-supplement, radiotherapy

## Abstract

Anticancer treatment is largely affected by the hypoxic tumor microenvironment (TME), which causes the resistance of the tumor to radiotherapy. Combining radiosensitizer compounds and O_2_ self-enriched moieties is an emerging strategy in hypoxic-tumor treatments. Herein, we engineered GdW_10_@PDA-CAT (K_3_Na_4_H_2_GdW_10_O_36_·2H_2_O, GdW_10_, polydopamine, PDA, catalase, CAT) composites as a radiosensitizer for the TME-manipulated enhancement of radiotherapy. In the composites, Gd (Z = 64) and W (Z = 74), as the high Z elements, make X-ray gather in tumor cells, thereby enhancing DNA damage induced by radiation. CAT can convert H_2_O_2_ to O_2_ and H_2_O to enhance the X-ray effect under hypoxic TME. CAT and PDA modification enhances the biocompatibility of the composites. Our results showed that GdW_10_@PDA-CAT composites increased the efficiency of radiotherapy in HT29 cells in culture. This polyoxometalates and O_2_ self-supplement composites provide a promising radiosensitizer for the radiotherapy field.

## 1. Introduction

Radiation therapy (RT), is one of the most commonly used treatments to inhibit tumor growth [1]. However, as many as 40% of tumors develop resistance to radiation therapy, significantly complicating treatment [2]. Tumor hypoxia is one of the important causes of radiation resistance [3]. The presence of hypoxic cells in tumor tissues is an important factor to reduce the effects of radiation exposure [4]. The oxygenated state of cells has a great influence on the killing effect of radiation, and the radiation has a strong killing effect on oxygenated cells, while the killing effect on hypoxic cells is obviously weakened [5]. It has been documented that cells irradiated under well-oxygenated conditions are 2–3 times more sensitive to radiation effects than hypoxic cells [6]. Tumor tissue often has problems of insufficient blood supply and a high ratio of hypoxic cells, and some cancer cells can escape radiation damage, which is one of the common reasons for tumor recurrence after radiotherapy [7]. Hypoxic cells in tumor tissues are resistant to chemoradiotherapy, and they continue to maintain survival function by anaerobic glycolysis. After the hypoxic state is improved, they can divide and proliferate, which becomes the root of tumor recurrence and metastasis in the future [8].

A radiosensitizer is a molecule/material that enhances the radiosensitivity of tumor cells [9]. Commonly, radiosensitizers are classified into five categories: (1) the inhibition of intracellular mercaptan or other endogenous radiation protective substances; (2) radiosensitizer radiolysis to form cytotoxic substances; (3) biomolecular repair inhibitors; (4) thymine analogues that can be incorporated into DNA; (5) oxygen simulants with electrophilic activity [10]. Many studies have found that nanoparticles synthesized by the atomic number (high-Z) elements have effective lethal effects on tumors [11,12,13,14,15]. When x-rays or γ-rays are applied to metal nanoparticles, a range of effects can occur. Photoelectron scattering produces special particles that enhance the activity [16]. Specifically, the energy that kills cancer cells is transferred from radiation waves to particles that act on subcellular structures, eventually causing fatal effects [17]. These nanoparticles interact directly with radiation by increasing its absorption or scattering, causing more local energy deposition, producing secondary electrons, alpha particles, Auger electrons, ionized, fluorescent photons, and free radicals [18,19,20]. Gold nanoparticles have been widely used in tumor radiosensitization due to their high atomic number, small size, easy accumulation in tumors, good biocompatibility, low toxicity, and relatively easy synthesis and combination of biological targets [14]. In addition, titanium, lanthanide-based nanoparticles, or cadmium selenide quantum dots, are also used for tumor radiosensitization [21,22,23,24]. With the continuous innovation of technology, more and more materials are defined as radiosensitizers [25]. Recently, it was found that polyoxometalates (POMs) are radiation enhancers by the aid of depositing more high-energy photons and secondary electrons into tumor tissue [26]. POMs are inorganic metal oxide clusters, which have many remarkable physical and chemical properties and biological activities [27]. The potential application fields of POMs cover almost all areas, including catalysis, photoelectric functional materials, and pharmaceutical chemistry [28,29,30]. For example, Yamase et al. reported that [NH_3_Pr*^i^*]_6_[Mo_7_O_24_]·3H_2_O (PM-8) had antitumor activity against human Co4 colon cancer, MX-1 breast cancer, and lung cancer [31]. In addition, the group also proposed the mechanism that a single electron reduction/oxidation cycle of PM-8 in tumor cells could inhibit the ATP generation. However, it is still limited in the field of POMs with high-Z elements as radiosensitizers.

In addition to depositing the radiation dose at the tumor site, increasing oxygen content in tumor cells is an important strategy for high-Z metal radiosensitizers [32]. These metal-based radiosensitizers are highly O_2_-dependent because O_2_ molecules can be used to generate numbers of destructive oxygen radicals as well as permanently immobilize ionizing radiation-induced biomolecule damage. Moreover, O_2_ can also reacts with the end of a DNA fracture to produce stable, hard-to-repair organic peroxides, greatly improving the degree of RT-induced cell damage [33]. However, the hypoxic TME impedes the RT. Recently, new materials are employed to alleviate tumor hypoxia through delivering exogenous O_2_, generating O_2_ in situ, and reducing hypoxia-inducible factor expression [32]. For example, Gu et al. synthesized a BiO_2_-x nanosheet with catalase activity to overcome hypoxia-induced radiation resistance and improve the efficacy of RT [34]. Liu et al. made use of the nanoscale Kirkendall effect and prepared hollow Bi_2_Se_3_ nanoparticles by a simple cation exchange method. These hollow Bi_2_Se_3_ nanoparticles were functionalized with polyethylene glycol, injected into PFC (Perfluorocarbon), and then loaded with oxygen to alleviate tumor hypoxia [33].

Based on these strategies, polydopamine-coated GdW_10_ nanoparticles (GdW_10_@PDA) were synthesized by a one-step hydrothermal method. Polydopamine (PDA) is an emerging biomimetic adhesive polymer that can be oxidized and self-polymerized by dopamine (DA) [35]. PDA can be easily deposited on the surface of various types of materials to prepare hybrid materials. The functions of PDA include immobilizing biomolecules as attachment layers, improving the properties of initial materials, reducing agents and stabilizers, etc. [36]. Therefore, catalase (CAT) is conjugated to the surface of GdW_10_@PDA to form the GdW_10_@PDA-CAT complex. Among them, Gd and W elements, as high Z elements, can deposit the radiation dose and improve the effect of radiotherapy. CAT can catalyze H_2_O_2_ overexpressed in the tumor microenvironment to generate oxygen, alleviate tumor hypoxia, and synergistically improve radiotherapy efficacy. Therefore, our work synthesized a new radiosensitizer that enhances tumor radiotherapy by reducing the tumor hypoxic microenvironment and the high Z effect.

## 2. Results and Discussion

### 2.1. Fabrication and Characterization of GdW_10_@PDA-CAT

**Scheme 1:**
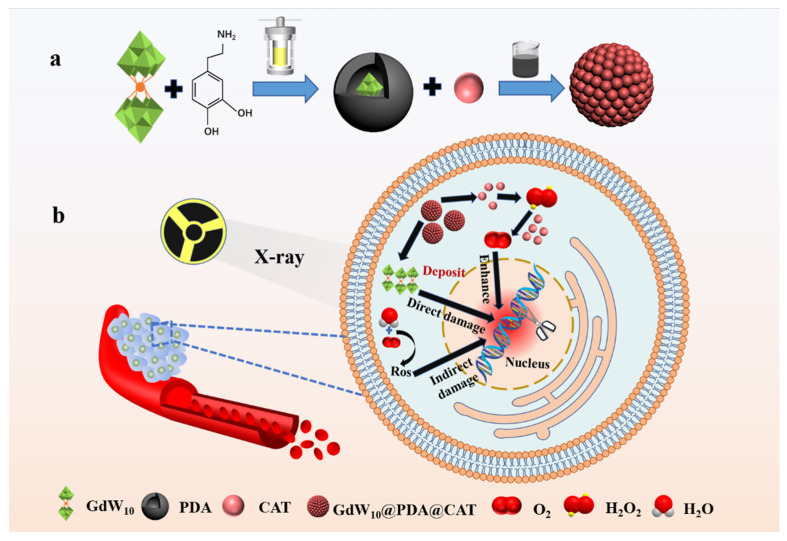
Schematic illustration of the sythesis process of GdW_10_@PDA-CAT (**a**) and the efficient radiosensitization efficacy of GdW_10_@PDA-CAT on tumor cells (**b**).

To engineer GdW_10_ and CAT composites, we chose to utilize the PDA as the conjugated group for its ability to coat different surfaces from inorganic molecular to biomolecular. As shown in Scheme 1a, dopamine was polymerized with GdW_10_ in situ by stirring to fabricate GdW_10_@PDA. The amino groups of DA could bond with the oxygen atoms of GdW_10_ through hydrogen bonding interaction. Then, CAT was immobilized on the surface of GdW_10_@PDA composites due to the adhesive ability of PDA with CAT, and, finally, GdW_10_@PDA-CAT was obtained. In this system, the high-Z elements Gd (Z = 64) and W (Z = 74) can deposit more X-ray energy and thus enhance radiation-induced DNA damages. The CAT of GdW_10_@PDA-CAT can catalyze H_2_O_2_ to produce O_2_ that alleviates tumor hypoxia and generates a number of destructive oxygen radicals (ROS) while stabilizing radiation-induced biomolecule damage. (Scheme 1b).

**Figure 1:**
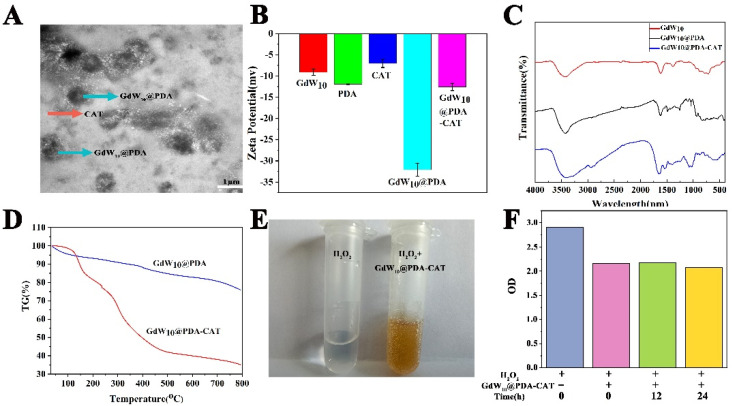
Characterization of GdW_10_@PDA-CAT. (**A**) TEM image of the as-synthesized GdW_10_@PDA-CAT. (**B**) IR spectra of GdW_10_, GdW_10_@PDA, and the as-fabricated GdW_10_@PDA-CAT. (**C**) Zeta potential analysis of GdW_10_, PDA, CAT, GdW_10_@PDA, and GdW_10_@PDA-CAT. (**D**) TG curve of GdW_10_@PDA and GdW_10_@PDA-CAT. (**E**) O_2_ production from the H_2_O_2_ solution catalyzed by GdW_10_@PDA-CAT. The solution: 10 mM H_2_O_2_ without/with 100 μg/mL GdW_10_@PDA-CAT at 25 °C, 10 min. (**F**) Absorption values of yellow complex formed through GdW_10_@PDA-CAT dispersed solution after adding H_2_O_2_ and Ti(SO_4_)_2_ over different periods of time.

In the literature, the sizes of POMs@PDA prepared from hydrothermal reaction range from 100 nm to 1.7 μm [37,38], whereas those micro-scale assembling particles with a size from 5 μm to 7.5 μm were synthesized under mild stirring conditions [39,40]. Small-sized hierarchical particles have a higher surface area and hence are better catalytic performers. Therefore, the hydrothermal condition was selected in the synthesis of the new GdW_10_@PDA composites. GdW_10_@PDA hybrids with different sizes were engineered by changing the GdW_10_ and DA mass ratio from 10:1 to 50:1, as shown in Figure S1A. Changing the mass ratio effectively changes the mean number-average diameters of the GdW_10_@PDA composites. It was found that the smallest size was about 100 nm when the GdW_10_ and DA mass ratio reached 25:1. Then, the amount of water was optimized based on keeping consistent with the GdW_10_ and DA mass ratio. The results showed that the particle size of GdW_10_@PDA was the smallest when the volume of water was 10 mL, as shown in Figure S1B. In the preparation of GdW_10_@PDA-CAT composites, the feeding ratio between GdW_10_@PDA NPs and CAT was 1:2. The TEM results (Figure 1A) exhibit the uniform solid microsphere of GdW_10_@PDA-CAT with a 606 ± 65 nm size. The Surface zeta potential measurements show that the ζ potential of GdW_10_ is decreased from −9.10 to −32.1 mV after polydopamine coating, and the ζ was increased from −32.1 to −12.6 mV after CAT modification. The increase 19.5 mV of ζ is consistent with an additional membrane onto the exterior of the GdW_10_@PDA core. When conjugated to CAT, the surface of GdW_10_@PDA-CAT becomes rough, as shown in Figure 1B.

Furthermore, in order to demonstrate the successful formation of GdW_10_@PDA-CAT, the FT-IR spectra of the GdW_10_, GdW_10_@PDA, and GdW_10_@PDA-CAT composites were performed (shown in Figure 1C). The characteristic asymmetric stretching vibration peaks of K_3_Na_4_H_2_(GdW_10_O_36_) 2H_2_O are found at 939, 874, 780, 716, and 503 cm^−1^, which were attributed to *ν*(W-O_terminal_) and *ν*(W-O_bridge_-W) of polyoxoanion. The characteristic peaks of K_3_Na_4_H_2_(GdW_10_O_36_) 2H_2_O were nearly consistent with those reported in the literature. The IR spectrum of GdW_10_@PDA retain the characteristic bands of GdW_10_ at 948, 896, 804, 644, and 531 cm^−1^ assigned to *ν*_as_(Gd-O_a_), *ν*_as_(W-O_d_), *ν*_as_(W-O_b_-W), and *ν*_as_(W-O_c_-W), indicating that the successful introduction of GdW_10_ into PDA and GdW_10_ retains the structure in composite materials. A typical absorption band at1429 cm^−1^ and a shoulder band at 1542 cm^−1^ are considered as C=C and C=O bonds in the aromatic ring, respectively, indicating the formation of quinone groups. These results clearly demonstrate the formation of PDA in GdW_10_@PDA. The spectrum of GdW_10_@PDA-CAT retain the characteristic peaks of GdW_10_ and CAT at 869, 772, 555, 424, 1451, 1401, and 2934 cm^−1^ assigned to *ν*_as_(Gd-O_a_), *ν*_as_(W-O_d_), *ν*_as_(W-O_b_-W), *ν*_as_(W-O_c_-W), *ν*_as_(COOH), and *ν*_as_(N-H). These peaks proved the formation of GdW_10_@PDA-CAT composites. The thermogravimetric analysis (TGA) results demonstrate that PDA is oxidatively decomposed between 250 and 500 °C. The content of GdW_10_ in the GdW_10_@PDA-CAT composite is calculated to be 34.5 wt% from TGA. It only lost 24.71% in GdW_10_@PDA (Figure 1D). In addition, we measured the content of GdW_10_ in GdW_10_@PDA-CAT composites by ICP-MS, and the results showed that the content of GdW_10_ was 504.8 μg, accounting for 5.88% of the content of GdW_10_@PDA-CAT.

For investigating the enzyme activity of CAT in the particles, H_2_O_2_ was added into the composites suspension and reacted for different times. As shown in Figure 1E, after adding GdW_10_@PDA-CAT composites into the H_2_O_2_ solution, a large number of bubbles were generated, indicating the generation of O_2_, which further proved the activity of CAT in GdW_10_@PDA-CAT. Then, the reaction was stopped by the addition of Ti(SO_4_)_2_ solution. The unreacted H_2_O_2_ could react with the added Ti(SO_4_)_2_ solution and form a kind of yellow complex, which could be detected via UV/vis at 410 nm according to the reported method. The enzyme activity of CAT was measured by monitoring the reduction of the yellow complex. As shown in Figure 1F, the redox reaction with CAT in the composites occurred immediately after the addition of H_2_O_2_. With the extension of time, the absorbance value at 410 nm remained unchanged, indicating that CAT had completely reacted; thus, the content of CAT could be calculated. The activity of catalase is 2000–5000 U/mg. 1 U refers to the decomposition of 1.0 μmol of H_2_O_2_ per minute under the condition of pH 7.0 and 25 °C. According to Lambert Beer law, the content of CAT in GdW_10_@PDA-CAT is calculated to be 10.24%.

The kinetics of GdW_10_ release from the composites under sink physiological conditions (37 °C, in large excess of 0.1 M PBS) were characterized. A time-dependent drug release study indicated that GdW_10_ was released in highly controllable manners. Approximately 30.17% of the GdW_10_ was released in the first 12 h and the 51.5% in 24 h. The PDA and CAT membranes had slower GdW_10_ release (Figure S2B).

### 2.2. The Cytotoxicity of GdW_10_@PDA-CAT

**Figure 2:**
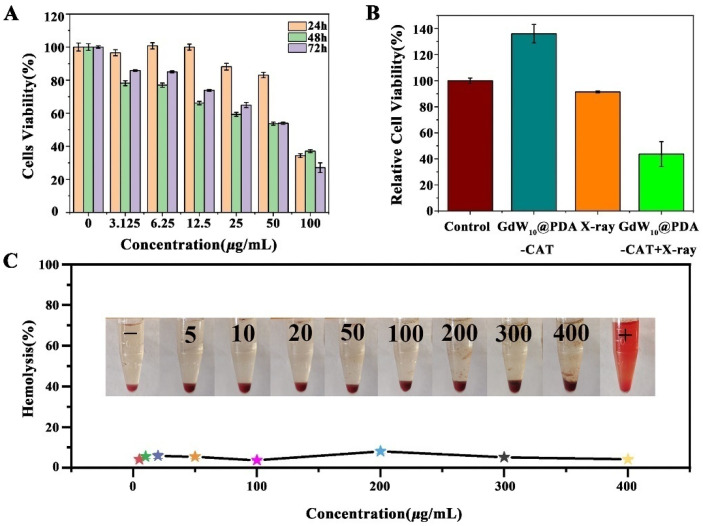
In vitro cytotoxicity evaluation of GdW_10_@PDA-CAT. (**A**) Cell viability of HT29 cells treated with GdW_10_@PDA-CAT of different concentrations for different times. (**B**) Vitality of HT29 cells after different treatments (GdW_10_@PDA-CAT: 50 μg/mL, X-ray: 6 Gy) for 24 h. (**C**) In vitro hemolysis rate of red blood cells incubated with different concentrations of GdW_10_@PDA-CAT. Deionized water (+) and PBS (−) acted as positive and negative controls, respectively.

In order to further explore the toxicity of nanoparticles to cells, the viability of HT29 cells was detected by MTT assay. HT29 cells were incubated with nanoparticles of different concentrations (0, 3.125, 6.25, 12.5, 25, 50, and 100 μg/mL) for different times (24, 48, and 72 h), and cell viability is shown in Figure 2A. The results showed that GdW_10_@PDA-CAT had low cytotoxicity. The cytotoxicity of GdW_10_@PDA-CAT on HT29 cells showed that the 50% lethal concentration (IC_50_) was about 81.03 μg/mL at 24 h, 48.86 μg/mL at 48 h, and 45.31 μg/mL at 72 h. After incubation for 24 h, the cells still showed high cell viability (above 80%) at 50 μg/mL. It was indicated that the composite particles presented good biocompatibility. Therefore, 50 μg/mL of GdW_10_@PDA-CAT composites were used in the following irradiation experiment. After 6 Gy irradiation treatment, the cell viability dramatically decreased in the presence of 50 μg/mL GdW_10_@PDA-CAT. It was decreased from 136.07% to 43.72%.

As shown in Figure 2B, when GdW_10_@PDA-CAT (50 μg/mL) and X-ray (6 Gy) were treated separately, the cell viability did not decrease, and the cell viability was 136.07% and 91.38%, respectively. However, the cell viability of the two combined treatments significantly decreased, and the relative cell viability was 43.72%. Therefore, GdW_10_@PDA-CAT presented obvious radiosensitization effects.

### 2.3. Hemolysis Test of GdW_10_@PDA-CAT

RBC hemolysis assay was used to evaluate the biocompatibility of the nanoparticles in vitro. Deionized water was used as the positive control and PBS as the negative control. The results showed that the GdW_10_@PDA-CAT particles almost did not cause hemolysis in the concentration range of 5–400 μg/mL (Figure 2C), which indicated that the GdW_10_@PDA-CAT particles had good blood compatibility.

### 2.4. Live/Dead Cell Staining

**Figure 3:**
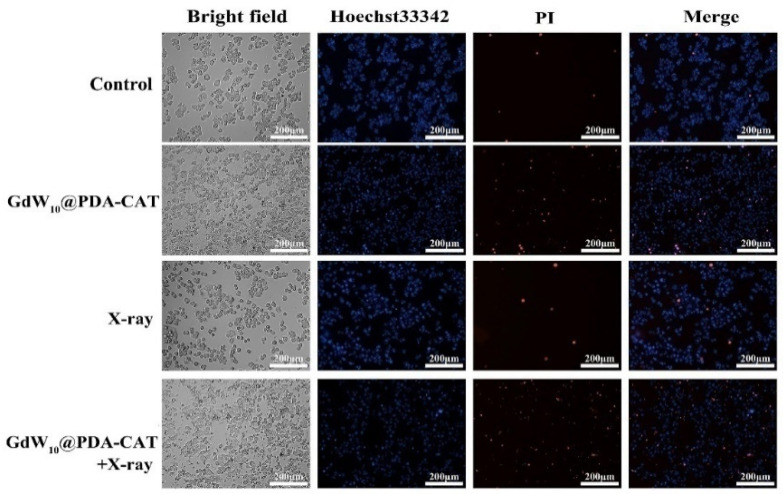
HT29 cells treated with GdW10@PDA-CAT (50 μg/mL) for 24 h and then stained with Hoechst33342 and PI to detect the Living/dead cells. Scale bar: 200 μm.

GdW_10_@PDA-CAT-induced apoptosis in HT29 cells was determined using a Hoechst33342/PI double staining assay. The color of cells can be stained into blue by Hoechst, while the nucleus can be stained into red by PI. Therefore, the normal cells were light blue, the apoptotic cells were brilliant blue and light red, and the dead cells were brilliant red. As shown in Figure 3, a small number of normal cells, apoptosis cells, and dead cells were observed in the control group. The GdW_10_@PDA-CAT and X-ray alone groups also had some apoptotic and necrotic cells, but a combination of the two showed obviously more cell death. It is proved that the GdW_10_@PDA-CAT particles can enhance the radiosensitivity of HT29 cell lines.

### 2.5. GdW_10_@PDA-CAT Particles Induced Death of HT29 Cells by Flow Cytometry

**Figure 4:**
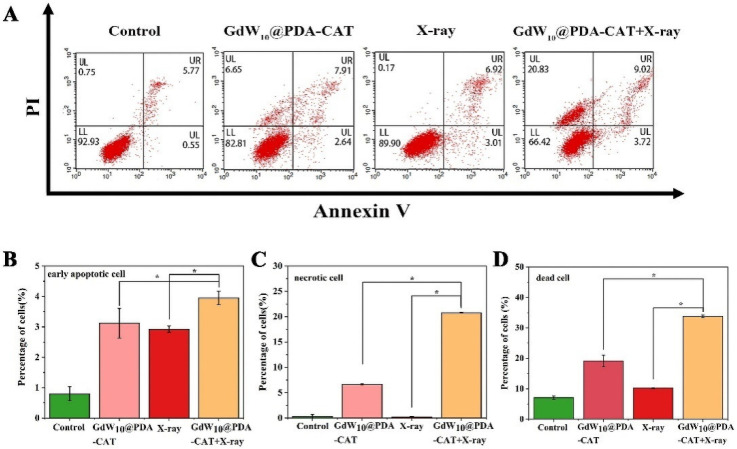
Apoptosis and necrosis of cells under different treatments. (**A**) Flow cytometric analysis of HT29 cell death under different treatments. Statistical analysis of early apoptotic cells (**B**), necrotic cells (**C**), and dead cells (**D**) under different treatments. The *p* Values were calculated by the Student’s *t* test: * *p* < 0.01.

To further confirm the effect of GdW_10_@PDA-CAT on the dead of HT29, the cells treated by GdW_10_@PDA-CAT for 24 h were double-stained with Annexin V/PI and analyzed using flow cytometry. As shown in Figure 4, both GdW_10_@PDA-CAT and X-Ray treatment alone led to significant apoptosis. When the two treatments acted on the cells together, the early apoptosis rate was more obvious, which was statistically different from that of the X-ray and GdW_10_@PDA-CAT particles alone. Cells treated with particles showed significant necrosis (6.65%) compared to cells treated with irradiation alone (0.25%). However, when the GdW_10_@PDA-CAT particles and X-ray were combined, cell necrosis was further aggravated, with a necrosis rate of 20.76%. This indicates that GdW_10_@PDA-CAT particles and RT have obvious synergistic effects. Therefore, it can be concluded that GdW_10_@PDA-CAT particles have radiosensitizing ability to improve the efficacy of radiotherapy for tumors.

### 2.6. Radiosensitizing Efficiencies of GdW_10_@PDA-CAT

**Figure 5:**
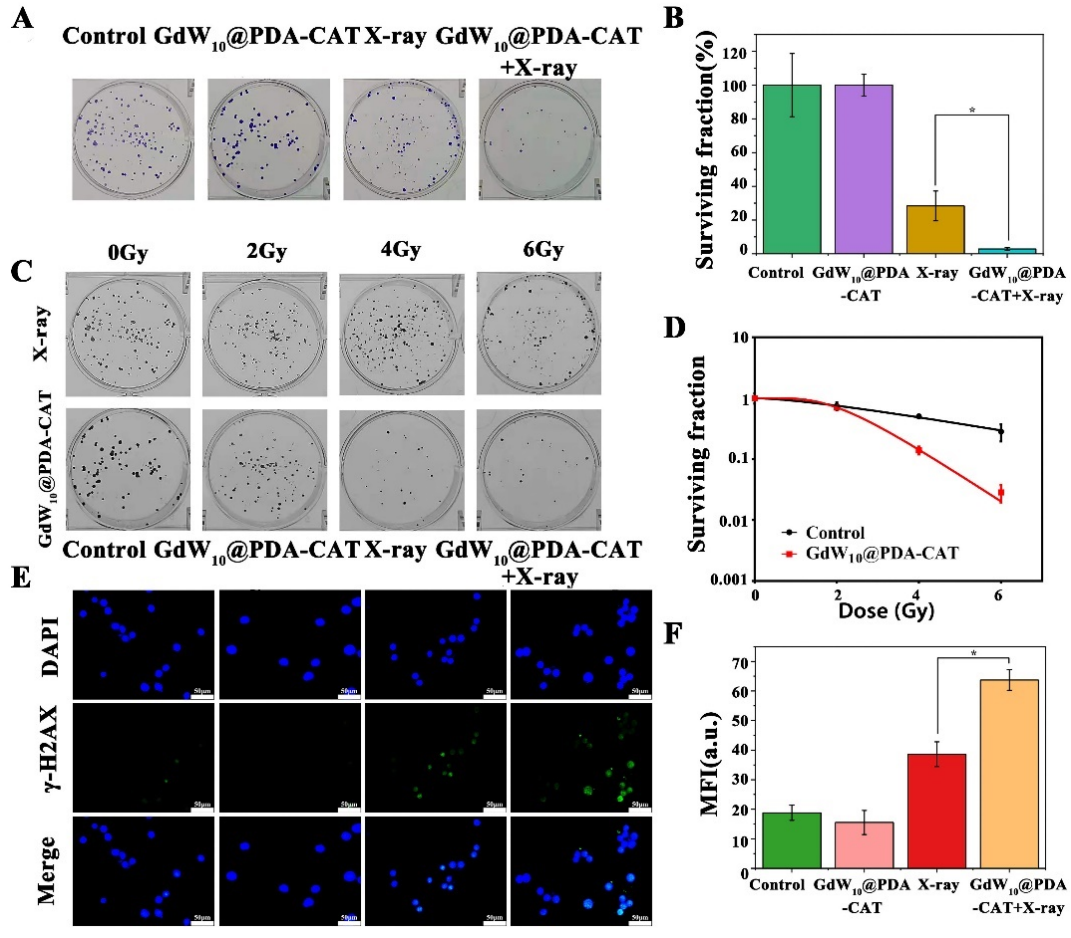
In vitro colony formation assay and DNA double-strand damage in HT29 cells. (**A**) Proliferation of HT29 cells under different treatments in vitro (GdW_10_@PDA-CAT: 50 μg/mL, X-ray: 6 Gy). (**B**) Survival fraction of HT29 cells after different treatments. (**C**) Colony formation analysis of HT29 cells treated with or without GdW_10_@PDA-CAT at different radiation doses. (**D**) Corresponding cell survival fraction after different treatments. (**E**) Representative immunofluorescence images of γ-H_2_AX with different treatments inducing DNA double strand breaks. The scale bar is 50 μm. (**F**) Quantitative analysis of FITC in cells with different treatments. The *p* Values were calculated by the Student’s *t* test: * *p* < 0.01.

The efficacy of GdW_10_@PDA-CAT was studied in HT29 cell lines via clonogenic survival assays, as shown in Figure 5. Figure 5A,B shows that HT29 cell lines treated with GdW_10_@PDA-CAT (50 μg/mL) could significantly enhance the RT efficacy. The cell survival fraction of RT group was 28.37%, and that of GdW_10_@PDA-CAT combined with the RT group was 2.85%. From the colony formation assay, the radiosensitztion performance of GdW_10_@PDA-CAT was dose-dependent (Figure 5C,D). The sensitization enhancement ratio (SER) of GdW_10_@PDA-CAT was calculated to be 3.7 in HT 29 cells.

### 2.7. Detection of DNA Double Strand Breaks In Vitro

H_2_AX phosphorylation at Ser139 (formation of γ-H_2_AX) is a marker of DNA double-stranded breaks (DSBs) and can therefore be used to monitor DNA repair after the action of different genotoxic stresses, including ionizing radiation, environmental agents, and chemotherapy drugs irradiation. The sites of DSBs can be visualized as focal sites of γ-H_2_AX using antibodies and immunofluorescence microscopy. In addition, positive staining for phospho-H_2_AX may indicate genomic instability and the telomere dysfunction in tumor cells. Herein, double-stranded DNA breaks of HT29 were assessed by γ-H_2_AX staining. As shown in Figure 5E, HT29 cells showed a weak γ-H_2_AX immunofluorescence signal after being co-incubated with the medium and the medium with GdW_10_@PDA-CAT suspensions for 24 h, while the cells were irradiated with X-ray and GdW_10_@PDA-CAT and X-ray groups had a more significant signal than the other two groups. However, the cells treated with the combination of GdW_10_@PDA-CAT composites and X-rays had a significantly higher level of γ-H_2_AX foci (*p*) than cells treated with them alone (Figure 5F). Therefore, the results indicated that GdW_10_@PDA-CAT leads to more DNA double strand breaks and radiosensitization capacity.

### 2.8. ROS in HT29 Cells

**Figure 6:**
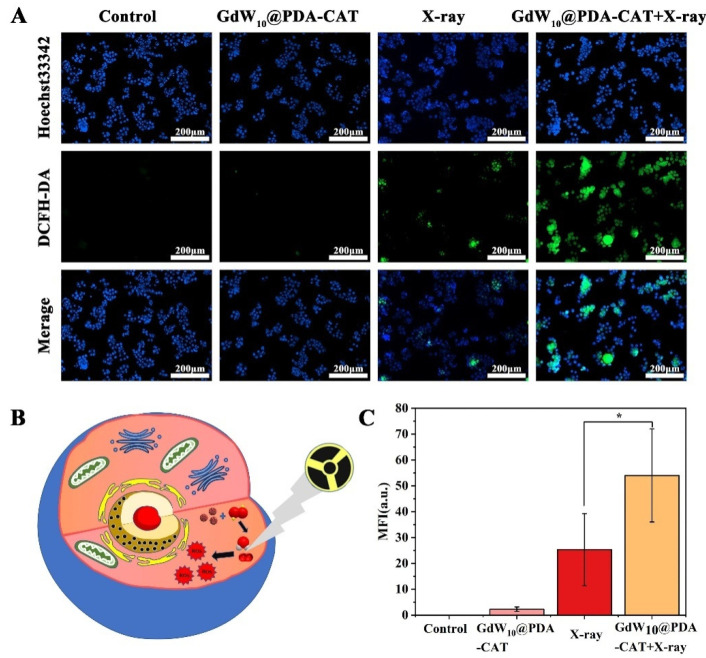
The production of ROS in vitro. (**A**) Fluorescence images for ROS generation in HT29 cells stained by Hoechst33342 and DCFH-DA after various treatments. The scale bar is 50 μm. (**B**) Schematic diagram of ROS produced by ionizing radiation. (**C**) Quantitative analysis of FITC in cells with different treatments. The *p* Values were calculated by the Student’s *t* test: *, *p* < 0.01.

The Hypoxic is a salient feature of solid tumors. Hypoxic tumors are often resistant to conventional chemotherapy, radiotherapy, and immunotherapy. Recently, research has concentrated on developing functional nanomaterials to treat hypoxic tumors. To elevate oxygen levels in tumors, oxygen-carrying nanomaterials, oxygen-generating nanomaterials, and oxygen-economizing nanomaterials have been used. It is well known that tumors have an acidic pH microenvironment and contain a high content of hydrogen peroxide (H_2_O_2_). CAT can convert H_2_O_2_ to O_2_ and H_2_O. The ionizing radiation of H_2_O will produce part of ROS to destroy tumor cells, while the production of O_2_ and H_2_O will increase the production of ROS [9,41]. The mechanism of ROS is shown in Figure 6B. Therefore, we fabricated an oxygen-generating material to elevate oxygen and H_2_O levels in tumors for enhanced radiotherapy. DCFH-DA was used as the probe to monitor ROS levels in HT29 cells treated with medium (control), GdW_10_@PDA-CAT, X-ray, and a combination of GdW_10_@PDA-CAT and X-ray. As shown in Figure 6A, the X-ray treatment group produced a strong fluorescence signal, while the irradiation combined with the GdW_10_@PDA-CAT treatment group showed a higher fluorescence signal (Figure 6C). The results are consistent with those described above. These results indicated that the GdW_10_@PDA-CAT composites had radiosensitizating ability through supplying O_2_.

## 3. Discussion

Radiation therapy (RT) is one of the most effective and frequent clinical cancer treatment methods [42,43,44]. The therapeutic principle of RT is that high-energy ionizing radiation (such as X-rays and γ-rays) directly interacts with cellular DNA to cause DNA damage (DNA is the main target that determines radiobiological effects), or indirectly reacts with water molecules to produce ROS (such as superoxide O_2_^−^, Hydrogen peroxide H_2_O_2_, hydrogen radical H, hydroxyl radical HO, and H_2_O^+^) to damage DNA or other cellular components and induce apoptosis and necrosis [45]. Although RT is widely used in the clinical treatment of tumors, it also has certain limitations. On the one hand, high energy ionizing radiation is easy to cause normal tissue damage and serious side effects [32]. On the other hand, the hypoxic microenvironment inherent in most malignancies results in tumor resistance to radiation during radiotherapy [46,47,48]. The GdW_10_@PDA-CAT composite herein served as a radiosensitizer to address the problems.

As high Z material, gadolinium and tungstate can absorb more X-rays [49]. Gu et al. reported the radiosensitization of Bi_2_WO_6_ on Hela cells in 2019 [50]. The γ-H2AX and colongenic assays demonstrated that PVP-Bi_2_WO_6_ could efficiently increase X-ray-induced intracellular DNA damages and colony formations. The results showed that the colony formation of Bi_2_WO_6_ and X-ray treatment group decreased to ~13.7%. In the same year, Yan et al. reported the radiosensitization effect of BiP5W30 on Hela cells. The result of cell colony formation indicates that the combination of X-ray and BiP5W30 significantly reduced survival to 25%. The sensitizer enhancement ratio of plus X-ray irradiation was calculated to be 1.41 [51]. You-Yeon Won et al. proposed the radiosensitization effect of CaWO4 on HN31 cells. It exhibited sensitizer enhancement ratios (SER) of 1.45 and 1.21 at a 10% survival fraction. Colongenic cell survival assay showed that CaWO_4_ exhibited a sensitization ratio (SER) of 1.21 at a 10% survival rate [52]. In 2017, Zhao et al. studied the radiosensitization effect of GdW10 on BEL-7402 cells, and the results showed that the SER was 1.28 [26]. Our results showed a similar enhancement of radiation efficacy. The Staining and Flow cytometry results showed that cells treated with GdW10@PDA-CAT in combination with X-ray showed more necrosis and apoptosis than those in the single treatment group. Colongenic survival assays showed that combined treatment significantly inhibited cell proliferation, and the SER was 3.7.

Studies have revealed that a direct improvement of the O_2_ level within tumors is the most effective approach to revere the radioresistance of hypoxia tumors. Some groups utilized perfluorocarbon (PFC) as a carrier to deliver exogenous O_2_ into tumors. For example, the PEG-Bi_2_Se_3_@PFC@O_2_ can result in a rapid O_2_ release under irradiation [33]. However, this strategy faces the problem of spontaneous O_2_ release from the O_2_ carrier before being internalized by cancer cells. Recently, given the large amount of H_2_O_2_ in the TME, the strategy of O_2_ generation in situ via catalyzing H_2_O_2_ provides a new way to produce O_2_ in situ. Catalase (a tetramer protein formed from a 60 kDa monomer unit) is an enzyme that catalyzes the decomposition of H_2_O_2_ into oxygen and water [53]. Our results show that the GdW_10_@PDA-CAT composite can generate a tremendous number of destructive oxygen radicals by catalyzing the H_2_O_2_ deposition as well as permanently immobilizing ionizing radiation-induced damage. From the staining, the combined X-ray and GdW_10_@PDA-CAT effectively enhances DNA double-strand breaks and the reactive oxygen species production. Therefore, the GdW_10_@PDA-CAT may be a potential agent to solve the tumor hypoxia and enhance the radiotherapeutic efficacy.

## 4. Materials and Methods

### 4.1. Materials

All of the chemicals were analytical grade reagents and without further purification. GdNO_3_·6H_2_O and catalase (CAT) from bovine live (≥40,000 units/mg protein) were purchased from J&K Scientific Ltd. (Beijing, China). Dopamine hydrochloride (DA), DCFH-DA, DAPI, Hoechst33342, and PI were supplied by Shanghai Aladdin Biochemical Technology Co., Ltd. (Shanghai, China). Na_2_WO_4_·2H_2_O was obtained from the Guoyao Chemical Research Institute (Shenyang, China). The HT29 cell line was obtained from the Cell Culture Center of the Institute of Basic Medical Sciences, Chinese Academy of Medical Sciences. 3-[4,5-di-methylthiazol-2-yl]-2,5-diphenyltetrazolium bromide (MTT) was purchased from Sigma-Aldrich (Shanghai, China). Annexin V-FITC apoptosis detection kit and Goat anti rabbit IgG-FITC were supplied by Absin Biotechnology Co., Ltd. (Shanghai, China). HIF-1α antibody and γ-H_2_AX antibody were purchased from Cell Signaling Technology, Inc. (Danfoss, CA, USA). McCoy’s 5A medium was obtained from Biological Industries (Shanghai, China). Fetal bovine serum was purchased from Sigma-Aldrich (Shanghai, China).

### 4.2. Characterization

The morphologies of the samples were observed using TEM (JEM-1011, Tokyo, Japan) with a working voltage at 100 kV and a scanning electron microscope (SEM, HITACHI S4800, Tokyo, Japan). The UV/Vis spectra were recorded on a TU 1810 UV-vis spectrophotometer (Beijing Persee General Instrument Co. Ltd., Beijing, China) or Microplate reader (BioTek, Winooski, VT, USA). A 1 cm path length quartz cuvette or 96-well microplates were used in the experiment. The Fourier Transform Infrared (FTIR) spectrum was recorded in the range of 400–4000 cm^−1^ on KBr (FTIR IR Affinity-1s, Shimadzu, Japan). Thermogravimetric analysis (TGA) was performed with a thermal analyzer at a heating rate of 10 °C min^−1^ in air (TGA 55, TA Instruments, New Castle, DE, USA). The pH measurements were performed by a PHS-25 pH meter (Shanghai INESA Scientific Instrument Co. Ltd., Shanghai, China). Particles’ size (nm) and surface charge (ζ-potential, mV) were characterized using a Zetasizer Nano Z dynamic light scattering detector (Malvern Instruments Ltd., Worcestershire, UK). A Heal Force Water Purification System was used (Shanghai Cantrex Analytic Instrument Co. Ltd., Pudong Shanghai, China).

### 4.3. Synthesis of K_3_Na_4_H_2_ (GdW_10_O_36_)·2H_2_O

K_3_Na_4_H_2_ (GdW_10_O_36_)·2H_2_O (GdW_10_) was prepared via a previously described method [26]. Briefly, Na_2_WO_4_·2H_2_O (8.3 g, 25 mmol) was dissolved in 20 mL of purified water set in a 50 mL beaker to form a uniform and transparent solution. Then, acetic acid was added to the aforementioned solution dropwise to adjust the pH to 7.3 under vigorous magnetic stirring at room temperature. Subsequently, 2 mL of Gd(NO_3_)_3_·4H_2_O (2.8 mmol) aqueous solution were added to the aforementioned solution dropwise, and a white precipitate was generated immediately. Then, 5 mL of KCl (3.2 mM) aqueous solution were added to the above solution, and it was stirred vigorously in a magnetic stirrer until the white precipitate disappeared and the solution became clear, at which point it was poured into another beaker and placed at 4 °C for a week. Crystals are produced overnight and dried at room temperature for use. The characteristic peaks of K_3_Na_4_H_2_ (GdW_10_O_36_)·2H_2_O (KBr pellet, cm^−1^) were 3425, 2384, 1613, 1471, 1386, 1167, 1120, 939, 874, 780, 716, and 503.

### 4.4. Preparation of GdW_10_@PDA Nanocomposites

125 mg GdW_10_ were dissolved in 7.5 mL water and stirred to transparency at 1000 rpm. Then, 5 mg DA were added. The mixture was stirred for 1 h at room temperature. The solution was transferred into a 15 mL Teflon-lined autoclave, which was maintained at 160 °C for 16 h. Black solution was obtained after cooling to room temperature. The obtained GdW_10_@PDA solution was purified by washing 3 times and concentrated using a 50 mL 100 kD ultrafiltration centrifuge tube (Millipore, Billerica, MA, USA) at 4000 rpm for 15 min. The free Na_8_H[α-PW_9_O_34_] in the product was separated, and the GdW_10_@PDA composites in the upper layer were re-suspended in deionized water for further experiments. The characteristic peaks of GdW_10_@PDA nanocomposites (KBr pellet, cm^−1^) were 3459, 2373, 1619, 1542, 1486, 1429, 1386, 1354, 1255, 1214, 1132, 948, 896, 804, 644, and 531.

### 4.5. Preparation of GdW_10_@PDA-CAT Composites

GdW_10_@PDA composites and CAT were mixed in the ratios of 1:2 in the PBS buffer (pH 7) with continuous stirring for 1 h at room temperature. The obtained GdW_10_@PDA-CAT was centrifuged at 3000 rpm for 10 min and washed several times with deionized water to get rid of the unreacted material. The product was concentrated to 1 mL. The purified GdW_10_@PDA-CAT composites were suspended in deionized water for future use. The characteristic peaks of GdW_10_@PDA-CAT (KBr pellet, cm^−1^) were 3438, 2934, 1644, 1541, 1451, 1401, 1029, 869, 772, 555, and 424.

### 4.6. Catalase Activity Detection

The enzyme activity of the GdW_10_@PDA-CAT was determined according to the previous method [41]. Each group of the 0.125 mg/mL GdW_10_@PDA-CAT aqueous solution samples was added with 1 mL H_2_O_2_ (10 mM). After 0, 12, and 24 h of reaction, 1 mL H_2_SO_4_ (25%) solution containing Ti(SO_4_)_2_ (0.6%) were added. Ti(SO_4_)_2_ could react with unreacted H_2_O_2_ to form yellow complex. The yellow complex can be detected with an ultraviolet spectrophotometer at 410 nm. In the control group, 1 mL H_2_O_2_ and 1 mL Ti(SO_4_)_2_ solutions were directly added without GdW_10_@PDA-CAT.

### 4.7. In Vitro Drug-Release Study

The in vitro drug release profiles of GdW_10_@PDA-CAT were recorded under sink conditions. GdW_10_@PDA-CAT solutions at a concentration of 0.5 mg/mL were split into Slide-A-Lyzer MINI dialysis microtubes with a molecular cutoff of 10 kDa (Pierce, Rockford, IL, USA) and subjected to dialysis against a large excess of phosphate buffer saline (200 μL of NP dispersion per 1 L of 1 M PBS) with gentle stirring at 37 °C. PBS was changed periodically (every 6–12 h) during the process. At the indicated times, 50 μL of GdW_10_@PDA-CAT solution were removed from the microtubes and mixed with 50 μL water and 900 μL of 6 M hydrochloric acid to dissolve the composites. Then, 5500 μL SnCl_2_, 200 μL KSCN, and 50 μL TiCl_3_ were added, respectively. GdW_10_ contents’ indifferent time points were determined at 400 nm using a TU 1810 UV-vis spectrophotometer and quantified using W standard curves.

### 4.8. Cell Culture

HT29 human colon adenocarcinoma cells were cultured using McCoy’s 5A medium supplemented with 10% (*v*/*v*) FBS and 100 U/mL penicillin-streptomycin in 5% CO_2_ and a humidified atmosphere of 95% at 37 °C.

### 4.9. In Vitro Cytotoxicity of GdW_10_@PDA-CAT Composites

The cytotoxicity effect of GdW_10_@PDA-CAT on HT29 cells was assessed by an MTT assay. Briefly, HT29 cells were seeded in 96 well plates at 10^4^ cells/well. Then, 12 h later, the cells were treated with GdW_10_@PDA-CAT composites at different concentrations ranging from 3.125 μg/mL to 100 μg/mL. Cells cultured without GdW_10_@PDA-CAT served as the control group. They were co-incubated for 24 h, 48 h, and 72 h at 37 °C, 5% CO_2_. For investigating irradiation, the cells were incubated with GdW_10_@PDA-CAT suspensions or medium for 24 h before irradiation. The cells were irradiated with 6 Gy using an X-RAD 320 (Precision X-ray) machine operating at 160 kVp and 25 mA. Then, 100 μL of MTT solution were added to each well and incubated for a further 3.5 h. Finally, 100 μL of DMSO were introduced for 20 min at 37 °C. Cell viability was then evaluated by measuring the optical density of 490 nm using a microplate reader (Biotek Co., Winooski, VT, USA).

### 4.10. Hemolysis Test of GdW_10_@PDA-CAT Composites

A Hemolysis test was used to evaluate the blood compatibility of GdW_10_@PDA-CAT composites. The whole blood of mice was centrifuged at 1500 rpm for 10 min under 4 °C and washed with saline to remove supernatant thrice. GdW_10_@PDA-CAT was added into 2% blood cells in the saline solution. DI water was added as 100% hemolysis. The mixtures were incubated for 2 h at 37 °C. The obtained solution was centrifuged to collect supernatant and to record the absorption of 540 nm. The hemolysis rate of each sample was determined by the following equation: Hemolysis % = [(sample absorbance − background absorbance)/(positive control − negative control)] × 100%.

### 4.11. Colony Survival Assay

Different numbers of HT29 cells at different densities of 200, 400, 800, and 1600 cells were seeded into 6-well plates and incubated for 12 h until they adhered to the well. After 24 h of growth, the cells were treated with the cell medium (control group), 50 μg/mL GdW_10_@PDA-CAT composites, a combination of 50 μg/mL GdW_10_@PDA-CAT composites and X-ray irradiation, or X-ray irradiation. The X-ray doses were 0, 2, 4, and 6 Gy for chemotherapy/chemoradiotherapy from an X-RAD 320ix (USA) machine operating at 180 kV and 20 mA. The dose rate was 1.020 Gy/min. The source-to-surface distance was 70 cm. The voltage and current were set as 180 kV and 20 mA, respectively, using a filter with a thickness of 1 mm, and the source-to-surface distance (SSD) was 70 cm. After 14 days of culture at 37 °C, the colonies were fixed in 4% (*v*/*v*) paraformaldehyde stained with crystal violet. All colonies with over 50 cells were counted. Experiments were performed in triplicate. The surviving fraction (SF) was calculated and analyzed using the single-hit multitarget model: SF = 1 − (1 − e^−D/D^_0_)^N^. SER values were calculated from the fitting of the experimental dose-damage curves for the fixed DNA damage effect by the formula: SER = D/D_drug_, where D is the dose for the observed effect in the control sample without GdW_10_@PDA-CAT and D_drugs_ is the dose evoking the same effect in the presence of GdW_10_@PDA-CAT [54].

### 4.12. Cell Morphological Observation

According to the previous study, Hoechst33342 (10 μg/mL) and PI (2.5 μg/mL) were used for morphological observation [55]. HT29 cells were seeded at a density of 1 × 10^5^ cells/mL in 96 well plates. After incubation for 12 h, cells were treated with 1 cell medium (control group), 50 μg/mL GdW_10_@PDA-CAT composites, a combination of 50 μg/mL GdW_10_@PDA-CAT composites and 6 Gy X-ray irradiation, or 6 Gy X-ray irradiation. The cells were further incubated for 24 h at 37 °C, 5% CO_2_. The cells were stained with Hoechst33342 and PI for 10 min at room temperature in the dark. The cellular morphology was observed using the fluorescence microscopy (Olympus Fluoview FV1000, Tokyo, Japan).

### 4.13. Flow Cytometry Assay

The apoptosis of HT29 cells was detected by a flow cytometer (FCM) using an Annexin-V FITC/PI kit purchased from Absin. According to the manufacturer’s instructions, HT29 cells were seeded in 6-well plates at a density of 1.5 × 10^5^ cells/mL and then treated with the cell medium (control group), 50 μg/mL GdW_10_@PDA-CAT composites, a combination of 50 μg/mL GdW_10_@PDA-CAT composites and 6 Gy X-ray irradiation, or 6 Gy X-ray irradiation for 24 h. Then, the cells were collected, washed with cool PBS and resuspended in 300 μL of binding buffer, incubated with 5 μL of FITC-conjugated annexin V for 10 min and 5 μL PI for 5 min at 37 °C away from light at room temperature, and then 200 μL binding buffer were added. For each treatment, at least 1.0 × 10^4^ cells were analyzed by a flow cytometer (BD AccuriC6, Franklin Lake, NJ, USA). The proportions in four phases were evaluated using BD analysis software.

### 4.14. Immunofluorescence of DNA Damage in HT29 Cells

HT29 cells were cultured on adherent cover slides in 6-well plates at a density of 1.5 × 10^5^ cells/mL for 12 h. The cells were treated with the cell medium (control group), 50 μg/mL GdW_10_@PDA-CAT composites, a combination of 50 μg/mL GdW_10_@PDA-CAT composites and 6 Gy X-ray irradiation, or 6 Gy X-ray irradiation. After 24 h of growth, the cells were fixed in 4% paraformaldehyde for 15 min and permeabilized with 0.05% Triton X-100 in DPBS for 10 min at room temperature. The cells were blocked with 5% BSA for 1 h at room temperature, then γ-H_2_AX rabbit monoclonal antibody (CST, Cat) for 1 h at room temperature. The cells were washed with PBS and incubated with the FITC tag goat anti-rabbit IgG secondary antibody (CST, Cat), diluted 1:200, for 1 h at room temperature. Nuclei were stained with DAPI. Images were acquired using a fluorescence microscopy (Olympus Fluoview FV1000, Tokyo, Japan). The number of γ-H_2_AX foci per cell was counted using Image J software.

### 4.15. Detection of ROS Generation in HT29 Cells

The ROS generating ability of GdW_10_@PDA-CAT composites was investigated by the 2′,7′-dichlorodihydro-fluorescein diacetate (DCFH-DA) assay. Briefly, HT29 cells were seeded on 96-well plates at a density of 1.0 × 10^5^ cells/mL. After 12 h of growth, the cells were treated with the cell medium (control group), 50 μg/mL GdW_10_@PDA-CAT composites, a combination of 50 μg/mL GdW_10_@PDA-CAT composites and 6 Gy X-ray irradiation, or 6 Gy X-ray irradiation. After 24 h of growth, the cells were gently washed twice with serum-free medium and stained with Hoechst33342 and a DCFH-DA probe in serum-free medium for 20 min at 37 °C. All operations were conducted under a dark environment and then washed three times with PBS. Afterwards, the cells were washed with PBS to remove unloaded DCFH-DA probe and were then imaged using a fluorescent image.

### 4.16. Statistical Analysis

The data were expressed as mean ± SD (standard deviation) and analyzed using the statistical package for the social sciences (SPSS, version 24.0, IBM SPSS, IBM Corp, Armonk, NY, USA). (Significance was established at *p* < 0.05.) All experiments were performed in triplicate. Statistical significance was evaluated by Student’s *t*-test.

## 5. Conclusions

In conclusion, our study has demonstrated that using high-Z effects and self-supplied oxygen is an excellent strategy to improve tumor radiotherapy. We engineered CAT decorated GdW_10_ encapsulated PDA composites that can enhance cell killing in HT29 cancer cell lines. The clonogenic survival assay demonstrated that the composites increased the efficiency of radiotherapy. Immunofluorescence studies revealed that GdW_10_@PDA-CAT efficiently deposited radiation doses and delayed tumor growth through the inhibition of DNA double-strand repair induced by radiotherapy. In addition, catalase in this composite was able to catalyze H_2_O_2_, which was generated by tumor metabolism, to produce O_2_. Future studies will focus on identifying other novel combinations of POMs and oxygen-supplied agents that are synergistic in radiotherapy.

## Data Availability

The data presented in this study are available in supplementary material.

## Supplementary Materials

The following supporting information can be downloaded. Figure S1: Size optimization. (A) Optimization of the mass ratio of GdW_10_ and DA. (B) Optimization of the volume of double distilled water. (C) Comparison of the particle sizes of GdW_10_@PDA-CAT synthesized with 7.5 mL and 10 mL water. Figure S2: The release of GdW_10_. (A) The standard curve of W. (B) Release rate of GdW_10_ in different time periods (1, 3, 6, 9, 12, 24, 48, 72, and 96 h).

## Sample Availability

Samples of the compounds are not available from the authors.
