# Supplementary material for: A GdW10@PDA-CAT Sensitizer with High-Z Effect and Self-Supplied Oxygen for Hypoxic-Tumor Radiotherapy
Source: Molecules. 2021 Dec 26;27(1):128. doi: 10.3390/molecules27010128 (PMC8746738; doi:10.3390/molecules27010128)
Supplement: Supplementary file 1 [file molecules-27-00128-s001.zip › molecules-1468795-supplementary.pdf]

molecules

Article

A GdW_{10}@PDA-CAT Sensitizer with High-Z Effect and Self-Supplied Oxygen for Hypoxic-Tumor Radiotherapy

Lixia Chen, Yang Zhang, Xinming Zhang, Ruijuan Lv, Rongtian Sheng, Ruimeng Sun, Ting Du, Yuhan Li and Yanfei Qi *

School of Public Health, Jilin University, Changchun 130021, China; chenlx20@mails.jlu.edu.cn (L.C.); yangzhang19@mails.jlu.edu.cn (Y.Z.); xmzhang19@mails.jlu.edu.cn (X.Z.); Lvrj20@mails.jlu.edu.cn (R.L.); shengrt19@mails.jlu.edu.cn (R.S.); Sunrm20@mails.jlu.edu.cn (R.S.); duting21@mails.jlu.edu.cn (T.D.); yhl21@mails.jlu.edu.cn (Y.L.)

* Correspondence: qiyanfei@jlu.edu.cn; Tel.: +86-431-8561-9441

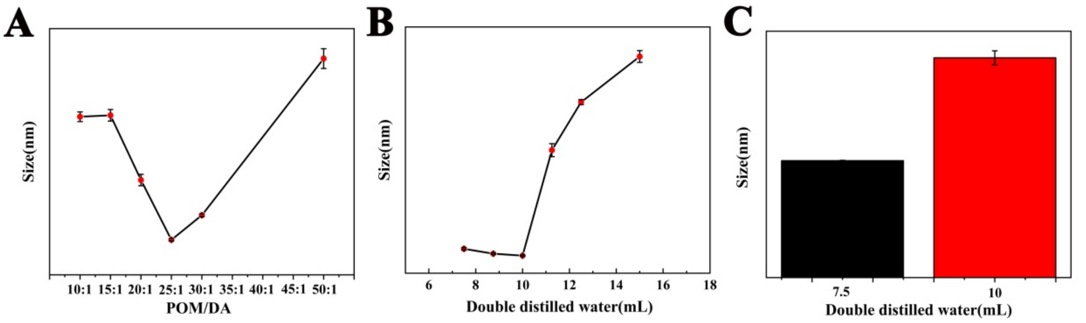

Figure S1. Size optimization. (A) Optimization of the mass ratio of GdW10 and DA. (B) Optimization of the volume of double distilled water. (C) Comparison of the particle sizes of GdW10@PDA-CAT synthesized with 7.5 mL and 10 mL water.

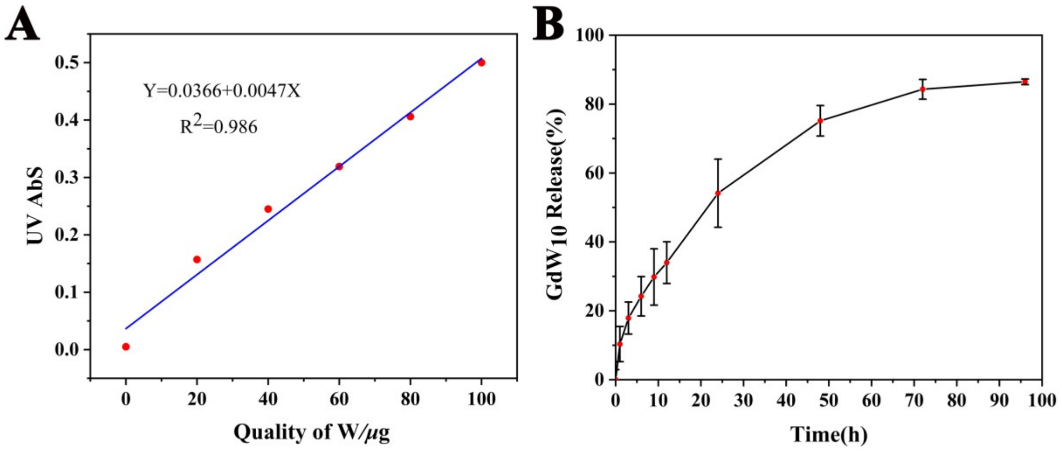

Figure S2. The release of GdW_{10}. (A) The standard curve of W. (B) Release rate of GdW10 in different time periods (1, 3, 6, 9, 12, 24, 48, 72, and 96 h).
